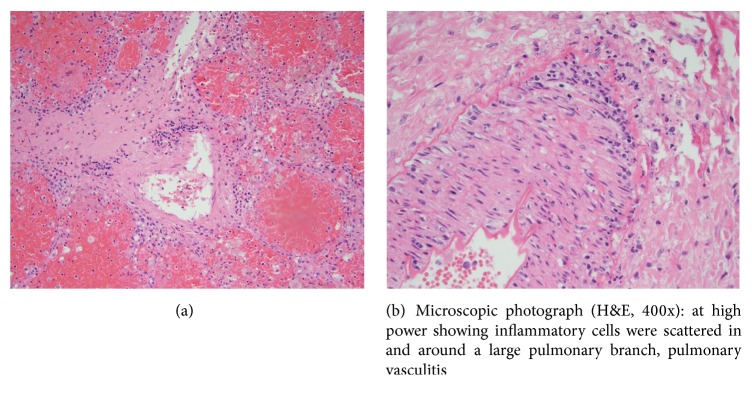# Pulmonary Vasculitis and a Horseshoe Kidney in Noonan Syndrome

**DOI:** 10.1155/2018/6829586

**Published:** 2018-01-29

**Authors:** Surasak Puvabanditsin, Rosanna Abellar, Adaora Madubuko, Rajeev Mehta, Lauren Walzer

**Affiliations:** ^1^Department of Pediatrics, Rutgers Robert Wood Johnson Medical School, New Brunswick, NJ, USA; ^2^Department of Pathology, Columbia University Medical Center, New York, NY, USA

## Abstract

We report a term male neonate with congenital myeloproliferative disorder, thrombocytopenia, a horseshoe kidney, feeding difficulty secondary to dysphagia/foregut dysmotility, and respiratory failure. Prenatal molecular genetic analysis revealed a fetus carrying* c.184T>G (p.Tyr62Asp)* pathogenic variant in* PTPN11*. The infant eventually succumbed to respiratory failure. Bacterial and viral cultures/studies were all no growth/negative. Pulmonary capillaritis and vasculitis were noted at autopsy. This report presents a new case of Noonan syndrome with unusual associated disorders and a review of the literature.

## 1. Introduction

Noonan syndrome (NS) is transmitted as an autosomal dominant trait and is genetically heterogeneous. So far, heterozygous pathogenic variants have been documented in seventeen genes (*PTPN11, SOS1, SOS2, KRAS, NRAS, RAF1, SHOC2, CBL, RRAS, RIT1, RASA2, MAP3K8, SPRY1, MYST4, LZTR1, A2ML1,* and* PP1CB*) that underlie this disorder or clinically related phenotypes [1, 2]. Pathogenic variants in PTPN11 gene (located on chromosome 12q24.1) are identified in approximately 50% of NS patients [3, 4]. NS is a variably expressed multisystem disorder with an estimated prevalence of 1 in 1000–2500 [3]. NS is characterized by short stature, congenital heart defect, and developmental delay. Other findings include webbed neck, pectus deformities, cryptorchidism, characteristic facies, coagulation defect, lymphatic dysplasias, and ocular abnormalities [4]. Pulmonary vasculitis and horseshoe kidney have not previously been reported in association with Noonan syndrome. We report a severe NS phenotype with a horseshoe kidney, thrombocytopenia, myeloproliferative disorder (MPD), feeding difficulty secondary to pylorospasm, and respiratory failure with pulmonary capillaritis and vasculitis. A review of the literature has been provided.

## 2. Case Presentation

A 3295 g male newborn was delivered at 38 weeks' gestation to a 34-year-old gravida 5 para 2 (2 miscarriages) Mexican mother by cesarean section secondary to prenatal diagnosis of NS fetus with possible thrombocytopenia. Apgar scores were 9 and 9 at 1 and 5 minutes, respectively. Pregnancy was complicated by a prenatal diagnosis of a large cystic hygroma at 16 weeks of gestation; amniocentesis was performed. The karyotype was 46, XY, and the single nucleotide polymorphism (SNP) microarray analysis was normal. Prenatal diagnosis of Noonan syndrome was made based on prenatal ultrasound findings and molecular genetic testing. There was no polyhydramnios noted on prenatal ultrasound. Family history was negative for congenital anomalies and consanguinity. There was no in utero exposure to known teratogens. Physical examination revealed a weight of 3295 grams (−0.25 *Z* score), length 44 cm (<−2 *Z* score), and head circumference 34 cm (−0.25 *Z* score). Multiple anomalies were noted: hypertelorism, low set ears, short philtrum, high arched palate, short neck, nuchal edema, and widely spaced nipples. Transient thrombocytopenia was noted during the first week of life; the lowest platelet count was 62,000/mm^3^. Leukocytosis (32–45 × 10^9^/L), monocytosis (8–28%), and blasts (2%) were noted throughout his 76 days of hospitalization. He had feeding difficulties (poor suck) and gastrointestinal symptoms (vomiting, abdominal distension) requiring gavage feeding since the first week of life. A contrast upper GI and small bowel follow-through study at 3 weeks of age showed delayed gastric emptying and pylorospasm. The baby did not thrive despite feeding with high calorie formula/breast milk. Abdominal and renal ultrasound showed a horseshoe kidney and a small splenic cyst (5 × 4 mm).

An echocardiogram on the second day of life revealed a secundum atrial septal defect, a moderate perimembranous ventricular septal defect, dysmorphic parachute mitral valve with mild mitral valve stenosis, dysplastic tricuspid valve, bicuspid aortic valve, and mild hypoplastic aortic arch. The infant's cardiac function gradually deteriorated, and at 5 weeks of age the baby required high supplemental oxygen via nasal cannula and diuretics. Repeat echocardiogram showed dilated right ventricle, tortuous right pulmonary vein, and pulmonary hypertension (PH). At 7 weeks of age, while waiting for cardiac catheterization to delineate the cause of PH, he developed respiratory failure that required extracorporeal membranous oxygenation (ECMO) for more than 3 weeks. Bacterial and viral cultures/studies were all unremarkable. At 11 weeks of age, cranial image revealed severe ischemic changes and parents agreed to withdraw life supporting measures.

## 3. Cytogenetic and Molecular Studies

The genes that were evaluated were* BRAF, CBL, HRAS, KRAS, MAP2K1, MAP2K2, NRAS, PTPN11, RAF1, and SHOC2*. The pathogenic variant NS gene* PTPN11* (NM 002834.3) was identified, genomic position: Chr12 (GRCh37): g.112888168, cDNA change: c.184T>G, amino acid change: p.Tyr62Asp.

## 4. Autopsy Findings

**Figure 1:**
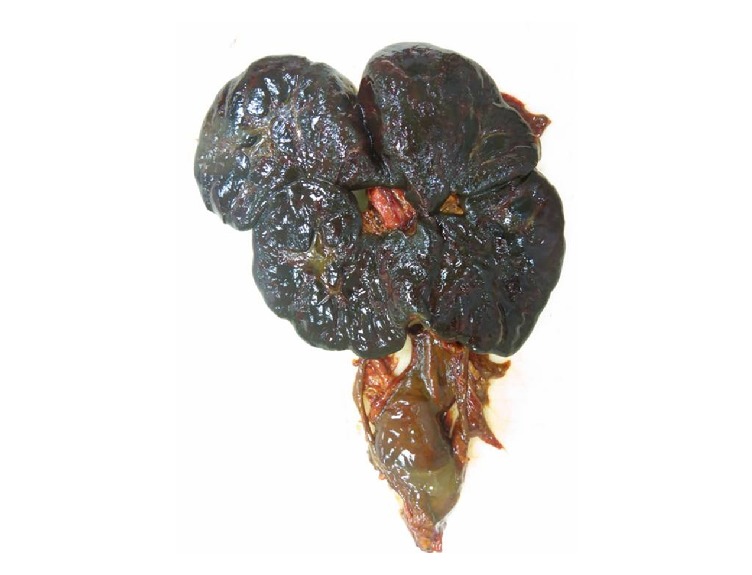
Photograph showing a horse shoe kidney.

External examination revealed dysmorphic features in keeping with Noonan syndrome. Internal examination confirmed a horseshoe kidney (Figure 1) with extensive extramedullary hematopoiesis. The cardiac examination showed moderate right ventricular hypertrophy, atrial septal defect (secundum type), perimembranous ventricular septal defect, dysplasia of mitral valve and tricuspid valve, bicuspid aortic valve, saccular dilatation of aorta, tortuous pulmonary artery, and patent ductus arteriosus. The bone marrow showed trilineage hematopoiesis with increased myeloid element. The myeloblasts and megakaryocytes were normal in number and morphology; M : E ratio was 6 : 1. Pulmonary capillaritis and vasculitis were noted at autopsy (Figures 2(a) and 2(b)).

## 5. Discussion

In 1963, Jacqueline Noonan first described nine NS patients with pulmonary valve stenosis, small stature, hypertelorism, mild intellectual disability, ptosis, undescended testes, and skeletal malformations [5]. Clinical features of NS include consequence of lymphatic obstruction/dysfunction during development, short webbing of the neck and prominence of the trapezius, cryptorchidism, widely spaced nipples, low set and posteriorly angulated ears, hypertelorism, downward slanting of palpebral fissures, and ptosis. Other main features are congenital heart defects (pulmonary stenosis, dysplastic pulmonary valve, hypertrophic cardiomyopathy, and secundum atrial septal defect), superior pectus carinatum with inferior pectus excavatum, developmental delay, short stature, and lymphatic dysplasias [3]. Congenital heart disease has been reported in 50–80% of NS patients; NS is the second most common syndromic cause of congenital heart disease, exceeded in prevalence only by trisomy 21 [3, 4, 6].

In 1994, linkage analysis of a large family inheriting NS established definitive linkage to the first NS locus, defined as chromosomal bands 12q22-qter and named NS. Genetic heterogeneity was subsequently established [7]. In 2001, Tartaglia and coworkers [8] identified missense mutations in the protein tyrosine phosphatase nonreceptor type 11 gene* (PTPN11)* as the first molecular causes of NS. This discovery was consolidated by the observations that* PTPN11* resided within the NS1 critical region and the studies with a mouse model of* PTPN11* deficiency revealed that its protein product,* SHP-2*, was critical for the embryologic development of the semilunar cardiac valves [9].

*PTPN11* pathogenic variants have been found in approximately 50% cases with NS and are also frequently positively associated with familial inheritance, pulmonary stenosis, ASD, HCM, short stature, sternal deformity, bleeding diathesis, juvenile myelomonocytic leukemia (JMML), and NS/MPD [3, 6, 10]. Only 1 study has provided an estimate of the frequency of the NS-related myeloproliferative disorder (NS/MPD) or monocytosis of NS [10]. The study demonstrated abnormal monocytic proliferation in 4 of 40 (10%) patients with NS, all occurring transiently during the neonatal period [11, 12]. The diagnosis of monocytosis of NS required high white blood cell count and peripheral blood smear exhibited notable monocytosis and absolute monocyte count > 1 × 10^9^/L. A monocytic proliferation disorder of NS that was noted in the early neonatal period may resolve spontaneously, persist with or without hepatosplenomegaly, or manifest as chronic myelomonocytic leukemia (CMML) [11]. Our case had NS/MPD or monocytosis of NS; the postmortem bone marrow excluded CMML. Pulmonary capillaritis and vasculitis findings in our case have not been reported with NS in the literature. Malignancies are among the well-established causes of vasculitis. Recently myeloproliferative disorders have been reported in association with vasculitis [13]. Vasculitis can affect any of the body's blood vessels. These include arteries, veins, and capillaries. Vasculitis may be a primary process, or it may occur secondary to a systemic infection, rheumatic or allergic disease, or malignancies including myeloproliferative disorders. Paraneoplastic vasculitis, immune pathogenesis including an associated cytokine transcriptional factor, interferon regulatory factor-1 [IFN-1], has been described in six patients with myeloproliferative disorders [14]. Leukocytoclastic vasculitis is the most common vasculitis associated with myeloproliferative disorders. Myeloproliferative disorders should be considered among disorders that are complicated by inflammation in the small blood vessels such as the capillaritis and vasculitis seen in our case. The pathogenesis still remains obscure.

Feeding difficulties occur frequently but are often unrecognized and cause major management difficulties in patients with Noonan syndrome. Feeding problems appear to be the result of delayed gastrointestinal motor development. However, the underlying etiology is poorly understood. The reported causes of feeding difficulties in NS patients were gastroesophageal reflux, delayed gastric emptying, and pseudo-obstruction syndrome [15]. The finding of pylorospasm in our case was new and supports previous reports of fore gut dysmotility in NS.

The prevalence of horseshoe kidney (HK) is approximately 0.25% in general population and 15% in females with Turner syndrome [16]. There were no reported cases of this unique genitourinary abnormality in NS. Gupta et al. (2009) reported a case of cross fused ectopic kidneys at the left lumbar region in a 14-year-old with physical features of Noonan syndrome [17]. In the report, the diagnosis was uncertain, and no mutation analysis or genetic study was performed on the patient. HK has not been reported in a patient with genetically (mutation analysis) confirmed Noonan syndrome.

## 6. Conclusion

In summary, we report a neonate with severe NS phenotypes including myeloproliferative disorder, transient thrombocytopenia, a secundum atrial septal defect, moderate perimembranous ventricular septal defect, dysplastic mitral and tricuspid valves, bicuspid aortic valve and mild hypoplastic aortic arch, pylorospasm, splenic cyst, and a horseshoe kidney. The molecular genetic analysis revealed a neonate carrying* c.184T>G (p.Tyr62Asp)* variant in* PTPN11*. The patient succumbed to complications of acute respiratory failure with pulmonary capillaritis and vasculitis. The pulmonary vasculitis and horseshoe kidney have not been previously reported in NS.

## Figures and Tables

**Figure 2:**